# Reconsidering hippocampal neurogenesis in Alzheimer's disease

**DOI:** 10.3389/fnins.2014.00147

**Published:** 2014-06-11

**Authors:** Alonso Martinez-Canabal

**Affiliations:** ^1^Molecular Neuropathology Department, Cell Physiology Institute, National Autonomous University of MexicoMexico City, Mexico; ^2^Cell Biology Department, Faculty of Sciences, National Autonomous University of MexicoMexico City, Mexico

**Keywords:** Alzheimer disease, hippocampal neurogenesis, hAPP, dentate gyrus, progenitor cells

Hippocampal neurogenesis is often thought to be necessary to maintain hippocampus-dependent cognitive abilities (see references in Deng et al., 2010). Most investigations using transgenic animal models of Alzheimer's disease (AD) report a reduction in hippocampal neurogenesis (see references in Mu and Gage, 2011) giving rise to the idea that impaired neurogenesis has an important role during the onset and progression of the disease. In many animal models of AD with familial-type mutations, this decrease in neurogenesis is associated with the presence of toxic amyloid beta peptides (Aβ_42_) (Haughey et al., 2002). Nevertheless, some works with transgenic animals have shown that amyloid deposition increases neurogenesis (Jin et al., 2004a; Lopez-Toledano and Shelanski, 2007; Yu et al., 2009). There was also a work with no conclusive results in this regard (Ermini et al., 2008). Still, the most general view in the field is that AD related neuropathology damages hippocampal neurogenesis and in consequence impairs cognition. Therefore, it is surprising that in a recent study published in *The Journal of Neuroscience*, Yetman and Jankowsky (2013) show that strong overexpression of mutated human amyloid precursor protein (hAPP) has no impact on hippocampal neurogenesis when hAPP expression excludes the proliferative region of the dentate gyrus.

Despite a large amount of data generated from studies employing animal models of AD, how hippocampal neurogenesis responds to AD in humans remains unclear. Some available data suggests that human AD is associated with a marked increase in the proliferation and survival of new neurons (Jin et al., 2004b; Perry et al., 2012). This works showed increased expression of neurogenesis markers not only during the onset but also during the middle and advanced stages of AD. Conversely Crews et al. (2010) reported a reduction in immature neurons during severe AD, although this data is not as comprehensive as the work Perry et al. (2012). Nevertheless, some researchers suggest that this effect is merely an artifact of disease-induced changes to endothelial cells (Boekhoorn et al., 2006), or that this new neurons may substitute for neurons lost due to AD (Kuhn et al., 2007; Baron et al., 2008).

Yetman and Jankowsky (2013) aimed to determine whether neurogenesis deficits observed in animal models of AD are due to changes intrinsic to progenitor cells, changes extrinsic to progenitor cells, or both. So, they generated a transgenic mouse model of AD in which mature glutamatergic cells overexpress mutant hAPP, resulting in the deposition of amyloid plaques formation only in the granule cell layer. After 6 months of gene activation, amyloid plaques appeared throughout the forebrain. In the dentate gyrus, many amyloid plaques were observed in the molecular layer and hilus but not in the granule cell layer or the proliferative zone. Furthermore, there were no changes in the level of hippocampal neurogenesis (Figure 1). This finding of unchanged neurogenesis differs radically from findings of reduced neurogenesis in other transgenic models in which amyloid protein production is not restricted to specific cell types. Consequently, Yetman and Jankowsky (2013) suggest that the neurogenesis deficits observed in other transgenic models are due to toxicity resulting from hAPP directly produced by progenitor cells and immature cells. Therefore, conflicts found in transgenic animals literature could be clarified exploring the patters of expression of hAPP. Humans with AD, however, do not exhibit reduced hippocampal neurogenesis (Perry et al., 2012). In addition, there is no evidence of APP expression in human neurogenic niche. Therefore, the transgenic mice used by Yetman and Jankowsky may be the currently existing animal model that most closely resembles human neuropathology.

**Figure 1:**
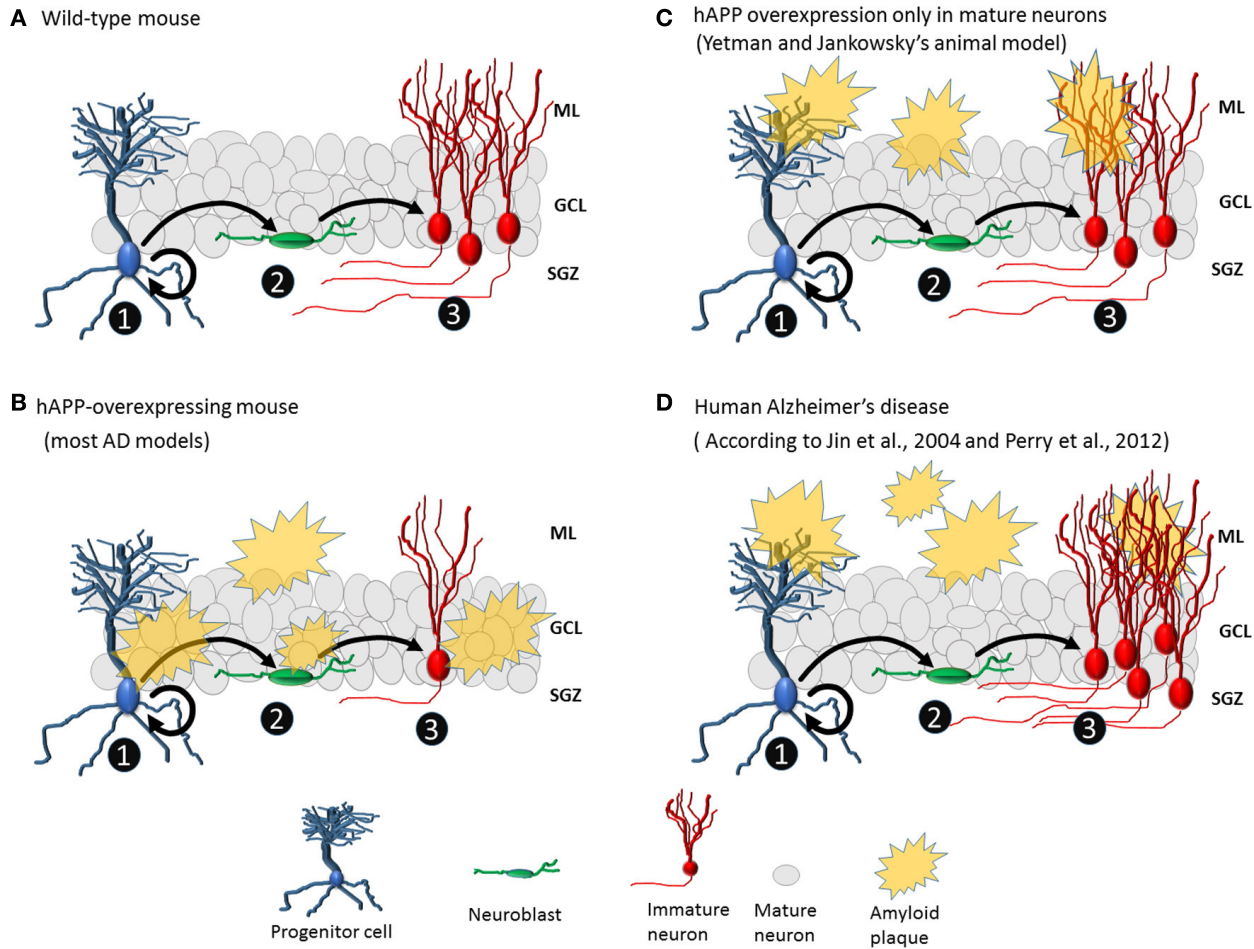
**Amyloid plaques affect neurogenesis differently in animal AD models and human AD. (A)** No plaques are present in wild-type mice. Progenitor cells divide asymmetrically, leading to the formation of new progenitor cells (1) and neuroblasts (2). Neuroblasts become immature neurons (3) that migrate to their final positions and extend dendrites and axons. **(B)** Common animal model of AD with hAPP overexpression and plaque formation in the granule cell layer and proliferative area of the dentate gyrus, which may directly affect the cell population from which new neurons are born. Normally, the number of immature neurons in transgenic mice is lower than that in wild-type mice. **(C)** In the animal model created by Yetman and Jankowsky (2013), plaques deposit mainly in the molecular layer of the dentate gyrus, and levels of neurogenesis are similar between transgenic and wild-type mice. **(D)** In human AD, there are plaques in the molecular layer but not around the granule cell layer or proliferative area of the dentate gyrus, and hippocampal neurogenesis is elevated (3).

The increase in hippocampal neurogenesis observed in humans with AD may possibly be due to disease-related inflammation. Variations in levels of inflammatory factors may affect neurogenesis by changing patterns of proliferation or survival of new cells. In particular, a strong inflammatory factor present in AD, transforming growth factor beta 1 (TGFβ-1), increases the number of granule neurons (Martinez-Canabal et al., 2013b) and enhances neurogenesis (Battista et al., 2006). Two other molecular players may also contribute to an increase in neurogenesis—hyper-phosphorylated tau (pTau) and mutated PS-1. Therefore, a hypothetical triple transgenic mouse (with hAPP, pTau, and PS-1 mutations) in which only hAPP is excluded from the dentate gyrus could provide clearer insights into how hippocampal neurogenesis is altered during AD. However, there might be several other unknown factors, both internal, related to molecular malfunction, or external, due to environmental effectors. The perfect model, closely resembling the real disease, seems to be challenging, but a closer approach is necessary to avoid the previous conflicts between existing models and the human disease regard neurogenesis.

Although the relevance of hippocampal neurogenesis to cognitive impairments in AD remains under debate, the assumption that disease-related neurogenesis loss is a key contributor to cognitive impairments could be fundamentally wrong. The evidence shows contradictory information about the aging decrease of neurogenesis and its impact on cognitive performance. Some studies support this view (Drapeau et al., 2003; Wati et al., 2006), but more recent works report no relation between neurogenesis decay with age and memory retention and retrieval (Merrill et al., 2003; Martinez-Canabal et al., 2013a,b). Therefore, there might be no reason for which age-related decreased neurogenesis implicates cognitive impairment. Rather than insufficient neurogenesis, excessive neurogenesis in pathological circumstances could lead to cognitive impairment by altering hippocampal circuits (Lee et al., 2012; Martinez-Canabal et al., 2013b). Therefore, to understand the role of hippocampal neurogenesis in AD-related memory impairment, we need additional transgenic models that exhibit neuropathology more similar to that occurring in humans. In addition, it is critical to understand if AD-associated neurogenesis yields properly connected and functional neurons that can support memory circuits. Immature neurons compared to mature, have different plastic characteristics that could lead to different memory roles. It would be important to understand the memory roles that neurons generated during AD if any, could develop (Ge et al., 2008). Although Yetman and Jankowsky's mouse model is close to the current needs of the field, we urgently need something closer, such as an animal model in which the expression of AD-related transgenes drives the production of new hippocampal cells.

## Conflict of interest statement

The author declares that the research was conducted in the absence of any commercial or financial relationships that could be construed as a potential conflict of interest.
